# Secondary infections worsen the outcome of COVID‐19 in patients with hematological malignancies: A report from the ITA‐HEMA‐COV

**DOI:** 10.1002/hon.3048

**Published:** 2022-08-12

**Authors:** Patrizia Zappasodi, Chiara Cattaneo, Virginia Valeria Ferretti, Roberto Mina, Andrés José María Ferreri, Francesco Merli, Margherita Oberti, Mauro Krampera, Alessandra Romano, Caterina Zerbi, Jacqueline Ferrari, Michele Cavo, Marco Salvini, Lorenza Bertù, Nicola Stefano Fracchiolla, Francesco Marchesi, Massimo Massaia, Vincenzo Marasco, Roberto Cairoli, Anna Maria Scattolin, Alessandro Maria Vannucchi, Carlo Gambacorti‐Passerini, Pellegrino Musto, Filippo Gherlinzoni, Antonio Cuneo, Antonello Pinto, Livio Trentin, Monica Bocchia, Sara Galimberti, Elisa Coviello, Maria Chiara Tisi, Alessandro Morotti, Brunangelo Falini, Mauro Turrini, Agostino Tafuri, Atto Billio, Massimo Gentile, Roberto Massimo Lemoli, Adriano Venditti, Matteo Giovanni Della Porta, Francesco Lanza, Luigi Rigacci, Patrizia Tosi, Sara Mohamed, Alessandro Corso, Mario Luppi, Nicola Giuliani, Alessandro Busca, Livio Pagano, Raffaele Bruno, Paolo Antonio Grossi, Paolo Corradini, Francesco Passamonti, Luca Arcaini, ITA‐HEMA‐COV Investigators

**Affiliations:** ^1^ Division of Hematology, Fondazione IRCCS Policlinico San Matteo Pavia Italy; ^2^ Division of Hematology Azienda Socio‐Sanitaria Territoriale‐Spedali Civili Brescia Italy; ^3^ Clinical Epidemiology and Biostatistic Service, Fondazione IRCCS Policlinico San Matteo Pavia Italy; ^4^ SSD Clinical Trial in Oncoematologia e Mieloma Multiplo Division of Hematology University of Torino Azienda Ospedaliero‐Universitaria Città della Salute e della Scienza di Torino Torino Italy; ^5^ Division of Hematology Istituto di Ricovero e Cura a Carattere Scientifico San Raffaele Scientific Institute Milan Italy; ^6^ Division of Hematology, AUSL‐IRCCS Reggio Emilia Reggio Emilia Italy; ^7^ Division of Hematology and Transplant Unit ASST Spedali Civili Brescia Italy; ^8^ Division of Hematology Azienda Ospedaliera Integrata di Verona Verona Italy; ^9^ Division of Hematology and Bone Marrow Transplantation Azienda Ospedaliera Universitaria Policlinico “G. Rodolico—San Marco” Catania Italy; ^10^ Department of Molecular Medicine University of Pavia Pavia Italy; ^11^ Division of Hematology Azienda Ospedaliero‐Universitaria Policlinico S. Orsola‐Malpighi Bologna Italy; ^12^ UOC Ematologia, Azienda Socio‐Sanitaria Territoriale Sette Laghi, Ospedale di Circolo of Varese Varese Italy; ^13^ Department of Medicine and Surgery University of Insubria Varese Italy; ^14^ Hematology Unit Fondazione IRCCS Ca' Granda Ospedale Maggiore Policlinico Milano Italy; ^15^ IRCCS Regina Elena National Cancer Institute Rome Italy; ^16^ Division of Hematology Santa Croce Hospital Cuneo Italy; ^17^ Division of Hematology Fondazione Istituto di Ricovero e Cura a Carattere Scientifico Istituto Nazionale dei Tumori University of Milan Milan Italy; ^18^ Hematology Azienda Socio‐Sanitaria Territoriale Grande Ospedale Metropolitano Niguarda Milan Italy; ^19^ Division of Hematology Azienda Unità Locale Socio‐Sanitaria 3 Serenissima Ospedale dell'Angelo Venezia‐Mestre Venice Italy; ^20^ Division of Hematology Azienda Ospedaliera Universitaria Careggi University of Florence Florence Italy; ^21^ Division of Hematology Ospedale S. Gerardo di Monza Monza Italy; ^22^ Department of Emergency and Organ Transplantation “Aldo Moro” University School of Medicine and Unit of Hematology and Stem Cell Transplantation, AOUC Policlinico Bari Italy; ^23^ Division of Hematology Ospedale Ca’ Foncello Treviso Italy; ^24^ Division of Hematology Azienda Ospedaliero Universitaria Arcispedale S. Anna Ferrara Italy; ^25^ Hematology, Istituto Nazionale Tumori Istituto di Ricovero e Cura a Carattere Scientifico “Fondazione G Pascale,” Naples Naples Italy; ^26^ Division of Hematology Azienda Ospedaliera di Padova Padova Italy; ^27^ Division of Hematology Policlinico Santa Maria alle Scotte Siena Italy; ^28^ Division of Hematology Azienda Ospedaliera Universitaria Pisana‐ Santa Chiara Pisa Italy; ^29^ Ematologia e terapie cellulari. IRCCS Ospedale Policlinico San Martino Genova Italy; ^30^ Division of Hematology Ospedale San Bortolo Vicenza Italy; ^31^ Department of Clinical and Biological Sciences University of Torino Orbassano Italy; ^32^ Division of Hematology and Transplant Unit Azienda Ospedaliera di Perugia Perugia Italy; ^33^ Division of Hematology Ospedale Valduce Como Italy; ^34^ Division of Hematology University Hospital Sant'Andrea Sapienza Rome Italy; ^35^ Division of Hematology and Transplant Unit Ospedale di Bolzano Bolzano Italy; ^36^ Division of Hematology Azienda Ospedaliera di Cosenza Cosenza Italy; ^37^ Department of Internal Medicine (DiMI) Clinic of Hematology, University of Genoa Genoa Italy; ^38^ IRCCS‐ San Martino Hospital Genoa Italy; ^39^ Department of Biomedicine and Prevention University Tor Vergata Rome Rome Italy; ^40^ Division of Hematology, Humanitas Clinical and Research Hospital Istituto di Ricovero e Cura a Carattere Scientifico and Department of Biomedical Sciences Humanitas University Milan Italy; ^41^ Division of Hematology Ospedale Santa Maria delle Croci Ravenna Italy; ^42^ Division of Hematology and Transplant Unit, Azienda Ospedaliera S. Camillo‐Forlanini Rome Italy; ^43^ Division of Hematology Ospedale degli Infermi Rimini Italy; ^44^ SC Ematologia Azienda Sanitaria Universitaria Giuliano Isontina Trieste Italy; ^45^ Division of Hematology Ospedale di Legnano Legnano Italy; ^46^ Department of Medical and Surgical Sciences UNIMORE. Division of Hematology Azienda Ospedaliero Universitaria Modena Modena Italy; ^47^ Division of Hematology and Transplant Unit Azienda Ospedaliero‐Universitaria di Parma S Italy; ^48^ Division of Hematology Fondazione Policlinico Universitario Agostino Gemelli ‐ Istituto di Ricovero e Cura a Carattere Scientifico Rome Italy; ^49^ Hematology, Università Cattolica del Sacro Cuore Rome Italy; ^50^ Division of Infectious and Tropical Diseases Fondazione Istituto di Ricovero e Cura a Carattere Scientifico Policlinico San Matteo Pavia Italy; ^51^ Department of Clinical Surgical, Diagnostic, and Paediatric Sciences University of Pavia Pavia Italy; ^52^ Division of Infectious and Tropical Diseases Azienda Socio‐Sanitaria Territoriale Sette Laghi, Ospedale di Circolo of Varese Varese Italy

**Keywords:** COVID‐19, hematological malignancies, outcome, secondary infections

## Abstract

The impact of secondary infections (SI) on COVID‐19 outcome in patients with hematological malignancies (HM) is scarcely documented. To evaluate incidence, clinical characteristics, and outcome of SI, we analyzed the microbiologically documented SI in a large multicenter cohort of adult HM patients with COVID‐19. Among 1741 HM patients with COVID‐19, 134 (7.7%) had 185 SI, with a 1‐month cumulative incidence of 5%. Median time between COVID‐19 diagnosis and SI was 16 days (IQR: 5–36). Acute myeloid leukemia (AML) and lymphoma/plasma cell neoplasms (PCN) were more frequent diagnoses in SI patients compared to patients without SI (AML: 14.9% vs. 7.1%; lymphoma/PCN 71.7% vs. 65.3%). Patients with SI were older (median age 70 vs. 66 years, *p* = 0.002), with more comorbidities (median Charlson Comorbidity Index 5 vs. 4, *p* < 0.001), higher frequency of critical COVID‐19 (19.5% vs. 11.5%, *p* = 0.046), and more frequently not in complete remission (75% vs. 64.7% *p* = 0.024). Blood and bronchoalveolar lavage were the main sites of isolation for SI. Etiology of infections was bacterial in 80% (*n* = 148) of cases, mycotic in 9.7% (*n* = 18) and viral in 10.3% (*n* = 19); polymicrobial infections were observed in 24 patients (18%). *Escherichia coli* represented most of Gram‐negative isolates (18.9%), while coagulase‐negative Staphylococci were the most frequent among Gram‐positive (14.2%). The 30‐day mortality of patients with SI was higher when compared to patients without SI (69% vs. 15%, *p* < 0.001). The occurrence of SI worsened COVID‐19 outcome in HM patients. Timely diagnosis and adequate management should be considered to improve their prognosis.

## 1 INTRODUCTION

Since the onset of the coronavirus disease 2019 (COVID‐19) pandemic, the mortality rate of patients with hematological malignancies (HM) infected by SARS‐COV 2 have been reported higher than in individuals without HM.^1^ Several studies have been published describing the clinical characteristics of COVID‐19 in HM patients and reporting an overall risk of death (ranging from 10% to 40%),^1^, ^2^, ^3^, ^4^, ^5^ substantially higher than in patients with solid tumors.^5^, ^6^, ^7^ Risk factors for mortality vary in different studies; however, COVID‐19 severity, presence of comorbidities, age, phase of treatment and status of progressive disease are reported as the most relevant factors impacting on outcome.^7^, ^8^, ^9^

It is known that infections of viral origin are often complicated by other pathogens able to impact the outcome. For instance, H1N1 influenza pandemic in 2009, but also other respiratory viruses, have been reported with variable percentage as associated to secondary pulmonary infections.^10^, ^11^

Additionally, in the current pandemic, secondary infections (SI) have been reported as a challenging issue influencing survival in critically ill patients.^2^

HM patients are intrinsically immunocompromized due to the underlying disease and to the treatment; moreover, the high rate of hospitalization besides chemotherapy induced‐neutropenia and impaired cellular‐mediated or humoral response increases the risk of additional infections.^3^, ^12^

There are some convincing data concerning the contribution of SI to worsen the outcome of COVID‐19 as reported in a comparison between survivors and non survivors of a large real‐life study on HM patients.^7^ However, little is known about the type and the evolution of SI during COVID‐19 in HM patients. The underlying immune system impairment can largely vary among diseases also in relation to the degree of immune control and to the status of the HM. With regards to the type of HM, acute leukemia patients have a higher risk of bacterial and mycotic infections, particularly in profound neutropenia periods and patients with lymphoproliferative diseases receiving immunotherapy depleting B‐ or T‐cell compartments can present viral reactivations.^5^, ^6^

In this ambispective study we present the available data on infectious events secondary to COVID‐19 in the large population of HM patients enrolled in the ITAlian HEMatology Alliance on COVid‐19 (ITA‐HEMA‐COV) project. The project already provided evidence on COVID‐19 mortality^7^ and seroconversion.^13^

With this analysis, we aimed to assess the impact of SI on the outcome of patients with HM and COVID‐19.

## 2 METHODS

### 2.1 Study design and participants

This multicenter, ambispective, cohort study involved 63 Hematology Units in Italy (see Appendix). The ITA‐HEMA‐COV group worked on behalf of all Italian scientific societies dealing with hematology: Società Italiana di Ematologia, Società Italiana di Ematologia Sperimentale, Gruppo Italiano Trapianto Midollo Osseo, Sorveglianza Epidemiologica Infezioni nelle Emopatie, and Fondazione Italiana Linfomi. We included consecutive adult HM patients (aged ≥18 years) admitted to hospital between 25 February 2020, and 31 March 2021 for COVID‐19, with data cut off for the analyses on 15 July 2021. Inclusion criteria were the symptomatic and laboratory‐confirmed SARS‐CoV‐2 infection, tested by RT‐PCR on nasopharyngeal swabs in patients with a WHO‐defined HM and the occurrence of microbiologically documented SI (bacterial, fungal, or viral). The study was approved by the institutional Review Board of each Hematology Unit. Written informed consent was collected from all patients except for those who were unable to give it (according to Italian law 9/2016 Autorizzazione Generale Garante della Privacy). This study is registered with ClinicalTrials.gov, NCT04352556, and the prospective part of the study is ongoing.

### 2.2 Procedures

Data on type and site of SI, laboratory parameters and patient outcome were collected for all participants since the date of the diagnosis of SI. Data on patient characteristics and outcomes were extracted by study investigators from electronic medical records or clinical charts, including age, sex, Charlson Comorbidity Index (CCI), type and status of HM, time since diagnosis of COVID‐19 to date of SI diagnosis and COVID‐19 severity.

Diagnosis of HM was made based on WHO classification of hematopoietic tumors.^14^ We defined a patient as having progressive disease in case of malignancy not responding to active therapy and remission as no evidence of disease; partial remission was defined as reduction of tumor burden during or after active treatment while stable disease if a stability during the time, without active treatment was present.

We categorized as “recently treated” patients who had received chemotherapy within the previous three months since COVID‐19 diagnosis, or immunotherapy/immunochemotherapy/biologic treatment within six months and as “on treatment” if the last hematological therapy was still ongoing at the time of diagnosis of COVID‐19.

All nasopharyngeal swabs for COVID‐19 diagnosis were managed according to national recommendations (https://www.iss.it/rapporti‐covid‐19/‐/asset_publisher/btw1J82wtYzH/content/id/5329985 ‐ 29 May 2020, accessed 4 Aug 2020).

Severity of COVID‐19 was graded according to the China Center for Disease Control and Prevention definitions^15^: mild (non‐pneumonia and mild pneumonia), severe (dyspnea, respiratory frequency ≥30 breaths per min, SpO_2_ ≤93%, PaO_2_/FiO_2_ <300, or lung infiltrates >50%), and critical (respiratory failure, septic shock, or multiple organ disfunction or failure).

SI were classified as ‘concomitant’ or ‘subsequent’ based on the interval within or over 48 h between diagnosis of COVID‐19 and of SI; based on the time when diagnosis of COVID‐19 was made, we considered two pandemic waves: the first wave from 20 February 2020 to 30 September 2020 and the second from 1 October 2020 to 31 March 2021.

Polymicrobial infection is defined when more than one type of organism were isolated from one or more blood cultures within a 72 h period. Bacteremia by coagulase‐negative staphylococci (CoNS) and by Corynebacteria was considered only when supported by at least two positive blood cultures. Gram‐positive and Gram‐negative isolates were considered multi‐drug resistant (MDR) according to Magiorakos et al.^16^ Proven or probable invasive fungal infections (IFI), classified according to EORCT criteria,^17^ were registered. We considered probable IFI as microbiologically documented if culture isolate or serum/bronchoalveolar lavage (BAL) galactomannan (GM) positivity was available. Information about antibiotic resistance profile of isolated bacteria were collected, if available.

### 2.3 Endpoints

The primary endpoint of this study was to define the incidence of SI in HM patients with a diagnosis of COVID‐19. Secondary endpoints were: (1) assessment of the association between patients' clinical characteristics at COVID‐19 diagnosis and the occurrence of SI; (2) description of all infectious events; (3) evaluation of the impact of SI on the 30 days‐mortality and on the overall survival (OS).

### 2.4 Statistical analysis

Qualitative variables were described as counts and percentage of each category. Quantitative variables were summarized as median and interquartile range (IQR). Association between two qualitative variables was tested via Fisher's exact test. Wilcoxon test was used to compare quantitative variables between two groups of patients. The cumulative incidence (reported together with its 95% confidence interval, 95%CI) was calculated from COVID‐19 diagnosis to secondary infection or last‐follow‐up and was estimated with a competing risks approach, analyzing death without infection as a competing event.

The association between baseline characteristics and the occurrence of SI was evaluated with univariable and multivariable Fine and Gray models. Variables with a *p*‐value<0.2 at univariable analysis were included in the multivariable model. Due to the collinearity between age and CCI, two models are presented.

OS was calculated from COVID‐19 diagnosis to death for any cause or last follow‐up; moreover, 30‐day mortality since COVID‐19 diagnosis was also assessed. The impact of the occurrence of SI on mortality was estimated with a pre‐defined multivariable Cox regression model, analyzing the occurrence of secondary infection as a time‐dependent covariate. Accounting for Bonferroni corrections, *p*‐values lower than 0.05 were considered significant. Statistical analyses were carried out with Stata 17 (StataCorp. 2021. Stata Statistical Software: Release 17. College Station, TX: StataCorp LLC).

## 3 RESULTS

### 3.1 Characteristics of the patients

A population of 1741 patients with HM and symptomatic COVID‐19 was enrolled in this study. Among them, 134 patients (7.7%) had a SI with a 30‐day cumulative incidence of SI of 5% (95%CI: 4.0%–6.2%).

The baseline characteristics of patients with or without SI are reported in Table 1. Overall, we found differences in the distribution of HM between patients with SI and those without SI (*P* < 0.001). Patients with SI have more frequently lymphoid neoplasms [non‐Hodgkin's lymphoma (NHL), Hodgkin's lymphoma (HL)] plus plasma cell neoplasms (PCN) (71.7% vs. 65.3%). Patients with SI were older (median age 70 vs. 66 years, *p* = 0.002), with greater CCI (median CCI 5 vs. 4, *p* < 0.001), with a higher rate of critical COVID‐19 (19.5% vs. 11.5% *p* = 0.046), and of uncontrolled disease (not in complete remission, CR: 75% vs. 64.7%). In SI patients there was a higher percentage of “recently treated” patients (50% vs. 31.4%, *p* = 0.001), with no difference for type of previous HM therapy (*p* = 0.887).

**TABLE 1:** Baseline characteristics of 1741 patients with hematological malignancy at the time of COVID‐19

	Total (*n* = 1741)	No secondary infection (*n* = 1607)	Secondary infection (*n* = 134)	*p*‐value
Gender, *n* (%)				0.237
Male	1018 (58.5)	933 (58.1)	85 (63.4)	
Female	723 (41.5)	674 (41.9)	49 (36.6)	
Age, median (IQR)	66 (55–75)	66 (55–75)	70 (62–76)	0.002
HM type, *n* (%)				<0.001
MPN	277 (15.9)	270 (16.8)	7 (5.2)	
MDS	94 (5.4)	88 (5.5)	6 (4.5)	
AML	134 (7.7)	114 (7.1)	20 (14.9)	
ALL/LL	57 (3.3)	54 (3.4)	3 (2.2)	
LYMPHOMA and PCN	1146 (65.8)	1050 (65.3)	96 (71.7)	
OTHER	33 (1.9)	31 (1.9)	2 (1.5)	
HM status, *n* (%)				0.024
CR	579/1676 (34.6)	548/1552 (35.3)	31/124 (25.0)	
Not in CR	1097/1676 (65.4)	1004/1552 (64.7)	93/124 (75.0)	
Charlson Comorbidity Index, median (IQR)	4 (2–6)	4 (2–6)	5 (3–7)	<0.001
Severity of COVID, *n* (%)				0.046
Mild	399/912 (43.8)	349/784 (44.5)	50/128 (39.1)	
Severe	398/912 (43.6)	345/784 (44.0)	53/128 (41.4)	
Critical	115/912 (12.6)	90/784 (11.5)	25/128 (19.5)	
Neutropenia, *n* (%)				0.289
Yes (<1000)	114/630 (18.1%)	95/545 (17.4%)	19/85 (22.4%)	
No (≥1000)	516/630 (81.9%)	450/545 (82.6%)	66/85 (77.6%)	
Recently treated, *n* (%)				0.001
Yes	335/1017 (32.9%)	293/933 (31.4%)	42/84 (50.0%)	
No	558/1017 (54.9%)	520/933 (55.7%)	38/84 (45.2%)	
On treatment	124/1017 (12.2%)	120/933 (12.9%)	4/84 (4.8%)	
Previous HM therapy, *n* (%)				0.887
Biologic compounds	127/335 (37.9%)	113/293 (38.5%)	14/42 (33.3%)	
Chemotherapy	97/335 (29.0%)	84/293 (28.7%)	13/42 (31.0%)	
Immunochemotherapy	76/335 (22.7%)	65/293 (22.2%)	11/42 (26.2%)	
Immunotherapy	35/335 (10.4%)	31/293 (10.6%)	4/42 (9.5%)	

*Note*: Recently treated NO: This category also includes: ‐
*patients treated with radiotherapy*. ‐
*patients who completed chemotherapy treatment more than 3 months before diagnosis of COVID‐19*. ‐
*patients who completed biologic/immunochemotherapy/immunotherapy treatment more than 6 months before diagnosis of COVID‐19*.

Abbreviations: AML, Acute Myeloid Leukemia; ALL/LL, Acute Lymphoblastic Leukemia; HM, Hematological malignancy; MPN, Myeloproliferative neoplasm, Myelodysplastic syndrome, PCN; Plasma Cell Neoplasm.

Univariable analysis found that advanced age, type of HM, higher CCI and COVID‐19 severity were significantly associated with the occurrence of SI (Table 2). Due to the collinearity between age and CCI, two multivariable models were built: in both models HM diagnosis was independently associated to occurrence of SI, with higher incidence of infection in AML, NHL and HL patients respect to MPN patients. No association was found with HM status and severity of COVID‐19 (Table 2).

**TABLE 2:** Univariable and multivariable association of baseline characteristics with the occurrence of secondary infection 1741 patients with hematological malignancy at the time of COVID‐19 (Due to the collinearity between age and CCI, two models are presented)

	Univariable analysis				Multivariable model 1				Multivariable model 2			
	sHR	95%CI	*p*‐value	Global *p*‐value	sHR	95%CI	*p*‐value	Global *p*‐value	sHR	95%CI	*p*‐value	Global *p*‐value
Age	1.02	1.01–1.03	0.001	0.001	‐	‐	‐	‐	1.0	1.0–1.0	0.786	0.786
HM				<0.001				<0.001				<0.001
MPN	Ref	‐	‐		Ref	‐	‐		Ref	‐	‐	‐
MDS	2.6	0.7–9.6	>0.90		4.4	0.8–23.9	>0.90		2.9	0.7–12.9	>0.90	
AML	8.0	2.9–21.5	<0.001		11.8	2.6–53.2	0.027		8.4	2.4–29.1	0.018	
ALL‐LL	3.1	0.7–12.8	>0.90		5.9	0.9–37.3	>0.90		4.0	0.8–20.8	>0.90	
LNH‐LH‐	4.9	2.0–12.2	0.014		9.1	2.2–37.9	0.051		6.4	2.0–20.6	0.041	
PCN	2.7	1.0–7.3	>0.90		5.4	1.2–24.1	0.568		3.5	1.0–11.9	>0.90	
HM status				0.108				0.721				0.502
CR	Ref	‐	‐		Ref	‐	‐		Ref	‐	‐	
Not in CR	1.4	0.9–2.2	0.108		1.1	0.7–1.8	0.721		1.2	0.7–1.9	0.502	
Gender				0.282				‐				‐
Male	Ref	‐	‐		‐	‐	‐		‐	‐	‐	
Female	0.8	0.6–1.2	0.282		‐	‐	‐		‐	‐	‐	
CCI	1.1	1.1–1.2	<0.001	<0.001	1.0	0.9–1.1	0.684	0.684	‐	‐	‐	‐
COVID‐19 severity				0.038				0.137				0.085
Mild	Ref	‐	‐		Ref	‐	‐		Ref	‐	‐	
Severe	1.0	0.7–1.6	>0.90		0.9	0.6–1.4	>0.90		1.0	0.7–1.6	>0.90	
Critical	1.9	1.1–3.2	0.051		1.7	0.9–3.0	0.290		1.8	1.0–3.1	0.131	
Neutropenia				0.582				‐				‐
No (≥1000)	Ref	‐	‐		‐	‐	‐		‐	‐	‐	
Yes (<1000)	1.2	0.7–2.1	0.582		‐	‐	‐		‐	‐	‐	

Abbreviations: AML, Acute Myeloid Leukemia; ALL/LL, Acute lymphoblastic Leukemia; CCI, Charlson Comorbidity Index; HM, Hematological malignancy; MPN, Myeloproliferative neoplasm, Myelodysplastic syndrome; PCN, Plasma Cell Neoplasm; sHR, sub‐Hazard Ratio from Fine&Gray model; 95%CI: 95% Confidence Interval.

When only patients “recently treated” or “on treatment” were considered, no difference in cumulative incidence of SI was found according to type of last HM therapy (*p* = 0.079), also after adjusting for line of therapy (*p* = 0.092).

### 3.2 Characteristics of secondary infections

Among 134 patients experiencing SI, a total of 185 microbiologically documented infectious events occurred with a rate of one infection per patient (IQR: 1‐1, range 1–8). In details, 110 patients experienced at least one bacterial infection (total number of bacterial events: 148), 17 patients experienced at least one fungal infection (total number of fungal events: 18) and 18 patients experienced at least one viral infection (total number of viral events: 19). Most fungal infections were recorded in lymphoproliferative diseases (17/18, 94%) and viral infections only in AML (3/19, 16%) and in lymphoproliferative diseases (16/19, 84%). Median time between COVID‐19 diagnosis and occurrence of SI was 16 days (IQR 5–36 days). Concomitant infections to SARS‐CoV2 were 39 (21%), while subsequent infections were 146 (79%).

Polymicrobial infection were observed in 24 (18%) patients. Gram‐negative bacteria infections were 72/148 (48.6%) and Gram‐positive were 76 (51.4%). *Escherichia coli* represented most of Gram‐negative isolates (28, 18.9% of all bacterial events), while CoNS were reported as prevalent Gram‐positive (21, 14.2% of all bacterial events). Isolates of SI are detailed in Supplemental Table 1. Antibiogram, available for 126 infections, reported antibiotic‐resistance in 48 cases (38%). In particular, methicillin and vancomycin resistance respectively accounted for 19/76 (25%) and 9/76 (11.8%) of Gram‐positive bacteria, Extended Spectrum Beta‐Lactamases (ESBL) producers and carbapenem‐resistant for 11/72 (15.3%) and 13/72 (18%) of Gram‐negatives, respectively (Table 3).

**TABLE 3:** Characteristics of 185 secondary infections in 1741 patients with hematological malignancy at the time of COVID‐19

	Bacteria *N* = 148	Fungi *N* = 18	Viruses *N* = 19
Time from COVID‐19 diagnosis, *n* (%)			
Concomitant	32 (21.6)	4 (22.2)	3 (15.8)
Subsequent	116 (78.4)	14 (77.8)	16 (84.2)
Isolate type, *n* (%)			
Bacteria			
Gram‐positive	76 (51.4)	‐	‐
Gram‐negative	72 (48.6)	‐	‐
Fungi			
Yeasts	‐	11 (61.1)	‐
Molds	‐	7 (38.9)	‐
Invasive fungal infection, *n* (%)	‐	18/18 (100)	‐
Antibiotic resistance, *n* (%)	48/126(38%)	‐	‐
Methicillin	19	‐	‐
Vancomycin	9	‐	‐
Carbapenems	13	‐	‐
ESBL producers	11		
Site of infection, *n* (%)			
Blood	78 (52.7)	13 (72.2)	9 (47.4)
Bronchoalveolar lavage	15 (10.1)	5 (27.8)	9 (47.4)
Urine	32 (21.6)	0 (0.0)	0 (0.0)
Feces	7 (4.7)	0 (0.0)	0 (0.0)
Skin	4 (2.7)	0 (0.0)	1 (5.2)
Other	12 (8.1)	0 (0.0)	0 (0.0)

As to the site of infection, most cases were bloodstream infections (Table 3), followed by cases documented in urine and bronchoalveolar fluid. Bacterial pneumonias were 40 (27% of bacterial infections). Grade 3–4 neutropenia was reported in 31 out of 169 infections (18.3%), in 27 out of 136 bacterial infections (19.9%), in 3 out of 15 (20%) mycoses and in 1 out of 18 (5.6%) viroses. Mycoses, mainly caused by yeasts (61.1%) were all invasive fungal infections, 7 probable aspergillosis (38.9%) and 11 proven candidemias (61.1%). Viral infections were 19 (12 Cytomegalovirus DNAemia, 2 Epstein Barr Virus DNAemia, 3 Varicella Zoster Virus, 1 Herpes Simplex Virus 1, 1 Rhinovirus). Lymphopenia was reported in 7 out of 18 cases at time of the viral infection (38.9%).

### 3.3 Impact of secondary infections on outcome

**FIGURE 1:**
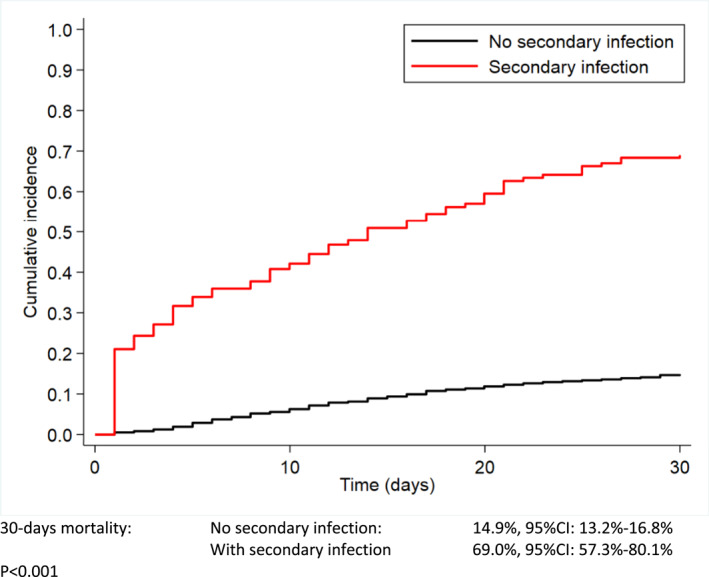
30‐day mortality of patients with and without secondary infection

**FIGURE 2:**
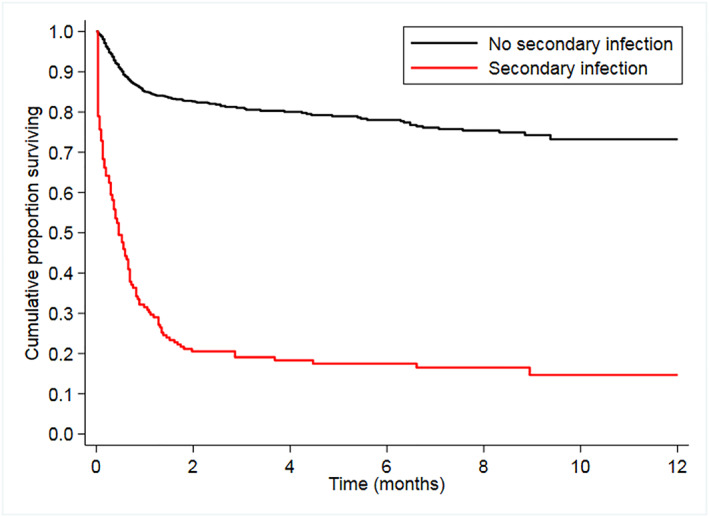
Overall survival of patients with and without secondary infection *P* < 0.001

Considering the entire cohort, after a median follow up of 1.5 months (IQR: 0.7–2.6 months), 362 patients died, 71 (53%) with SI and 291 (18.1%) without SI (HR = 6.7, 95%CI: 5.1–8.7, *p* < 0.001). The 30‐day mortality rate since COVID‐19 diagnosis was significantly higher in SI group (69% vs. 14.9%, HR = 6.5, 95%CI: 4.7–9.0, *p* < 0.001) (Figure 1). Median OS in patients with SI was 14 days compared to those without (not reached) (*p* < 0.001) (Figure 2). In a pre‐defined multivariable Cox model, after adjusting for age, COVID‐19 severity, status of HM, type of HM, OS resulted lower of almost 3 times in patients with SI compared with those without SI (HR = 2.9, 95%CI: 2.2–3.9, *p* < 0.001) (Supplemental Table 2).

Proportion of deaths at 30 days among patients having at least one bacterial infection was 36% (40/110) (at least one Gram‐negative: 17/62, 27.4%; at least one Gram‐positive 25/62, 40%). Proportion of deaths among patients with at least one mycosis was 23% (4/17) and among patients with at least one virosis, it was 22% (4/18). While a significant impact on 30 days‐mortality was evident if at least one bacterial infection (HR = 5.3, 3.9–7.2; *p* < 0.001) or one mycosis (HR 2.3, 1.0–5.3; *p* = 0.044) or one virosis (HR = 2.9, 1.5–5.9; *p* = 0.002) was present, no 30 days‐mortality difference was found by comparing bacterial versus fungal (*p* = 0.079), bacterial versus viral (*p* = 0.165) or fungal versus viral infection (*p* = 0.663). No significant difference in OS of patients with SI in the two different waves of the pandemic was observed (*p* = 0.328).

## 4 DISCUSSION

This study describes the findings on 134 patients with laboratory confirmed SI identified in a cohort of 1741 COVID‐19 patients with HM, showing an overall incidence of 7.7%. A similar incidence has been reported in large series of COVID‐19 patients from the general population with higher rates in studies including ICU patients.^2^, ^18^, ^19^

Scarce data are available on SI in HM patients; Gudiol et al.^8^ evaluated 684 patients with solid cancer and HM and reported a SI incidence of 17.6% in 300 HM patients. This high incidence can be partially explained by the enrollment criteria of infections: in our study we included only laboratory‐confirmed cases while Gudiol et al.^8^ described also infectious events suggested by clinical and radiological parameters without the need for microbiological confirmation. As a consequence, this can result in a possible higher estimation of SI; in the absence of rigorous required criteria, as recently suggested in large reviews and metanalyses,^19^, ^20^ it may be hard to distinguish complications caused by SARS‐CoV2 or by other possible not documented microbiological infections, due to the complexity of COVID‐19 pulmonary and extrapulmonary manifestation.

In our study, HM patients with SI were older, with a worse performance status and, in most cases, their HM was not in remission; on the other side, as already reported, the diagnosis of AML and NHL‐HL‐PCN were the prevalent conditions exposing to SI.^3^, ^5^

SI were more frequent in recently treated patients; however, we did not find a type of treatment favoring SI more than the others.

As expected and in line with the literature,^8^ infections developed in most cases after 48 h from admission. They were mainly bacterial with a similar incidence of Gram‐negative and Gram‐positive germs and the type of isolates did not impact on survival. Gram‐positive bacteria were mainly represented by CoNS, probably because of the severity of the disease often requiring central venous catheter. The finding of high frequency of Gram‐negative isolates is in line with the most recent epidemiologic reports observed in HM patients.^8^, ^21^ Fungal infections were all invasive and they represented 9.7% of all SI, more than reported by Garcia‐Vidal et al.^2^ However, an increase of IFI in HM patients has been recently reported by retrospective nationwide or single center studies,^22^, ^23^ mainly in neutropenic acute leukemia patients. Indeed, most of IFI observed in our cohort were detected in lymphoproliferative diseases or PCN, suggesting that probably other predisposing factors, like previous steroid treatment or humoral/cellular immunity impairment, may be involved. Mortality due to mycoses was very high, in line with data reported outside COVID19 scenario.^24^, ^25^, ^26^ Nonethless, mortality was not different according to the type of SI.

Although the respiratory tract was mainly involved in COVID‐19 infection, additional pulmonary infections were not prevalent. On the contrary, bloodstream infections represented the prevalent site of infection, mainly for bacteria. Therefore, bacterial pneumonia representing the 27% of all bacterial infections suggest a possible pulmonary involvement secondary to a bacteremia. The same mechanism can be at least considered for viral pneumonia mainly due to a reactivation of CMV.

COVID‐19 HM patients present poor outcomes with a higher mortality than in the general population or in solid cancer patients.^1^, ^2^ We previously reported that patients experiencing additional complications to COVID‐19, often represented by SI, have a worse prognosis as compared to patients without them.^7^

The present study specifically evaluated characteristics and impact on outcome of the additional infectious events complicating COVID‐19. We confirm that SI impact severely on outcome, inducing a 30‐day mortality rate more than 4 times higher than in HM patients without SI. In particular, 69% of patients with SI have died compared to 14.9% of HM patients without SI and median overall survival resulted very short. The occurrence of SI maintains its independent value on survival with age, COVID‐19 severity, and status of HM. This fatality rate is higher than in the general population with COVID‐19 where SI increase the risk to almost 10% compared to general COVID‐19 population without additional infections.^2^ It is also higher than that reported by Gudiol et al.^3^ who found a fatality rate of 34% in a similar setting of patients of HM patients with SI, surprisingly not so far from mortality rates reported in HM patients as a whole, regardless of additional infections.^1^, ^5^, ^9^

Our study has some limitations. First, due to its partial retrospective nature, information about characteristics of the infections were often heterogeneous among centers. To maintain a high level of accuracy of the analysis we collected only cases with a proven diagnosis, and we excluded all clinical diagnoses and all suspected cases for bacterial colonization based on the type of the pathogen identified or to the site of isolation. This selection could have determined an underestimation of the true incidence of the SI; we can reasonably assume that many infections have never been diagnosed and, therefore, we describe only a part of the true picture of infectious complications in HM COVID‐19 patients. Moreover, even if SI mainly developed after hospital admission we cannot exclude that a number of patients developed SI without being hospitalized. Another possible limit, also observed by other authors,^19^ could be the lack of available complete and consecutive information of all infectious complications, particularly during the first wave, immediately after the pandemic outbreak, due to the hard management of the health care system.

In conclusion, SI severely impact on survival of COVID‐19 HM patients; therefore, in these patients, from the early phases of COVID‐19, great attention should be paid to diagnosing a potential SI and to starting treatment promptly to minimize the risk of death.

## AUTHOR CONTRIBUTIONS

Luca Arcaini, Patrizia Zappasodi, Chiara Cattaneo, conceived the study, Patrizia Zappasodi and Chiara Cattaneo contributed to study design, study supervision, and data interpretation and wrote the paper. Virginia Valeria Ferretti and Lorenza Bertù, did the statistical plan, Virginia Valeria Ferretti, performed the analysis and interpreted the data. Luca Arcaini, Francesco Passamonti, Paolo Corradini, Livio Pagano, Paolo Antonio Grossi, Atto Billio, Roberto Mina, contributed to the interpretation of data analysis. Patrizia Zappasodi, Chiara Cattaneo, Luca Arcaini, Roberto Mina, Andrés José María Ferreri, Francesco Merli, Margherita Oberti, Mauro Krampera, F.D.R, Caterina Zerbi, Jacqueline Ferrari, Michele Cavo, Marco Salvini, Nicola Stefano Fracchiolla, Francesco Marchesi, Massimo Massaia, P.G, Roberto Cairoli, Raffaele Bruno, Alessandro Maria Vannucchi, Carlo Gambacorti‐Passerini, Pellegrino Musto, Filippo Gherlinzoni, Antonio Cuneo, Antonello Pinto, Livio Trentin, Monica Bocchia, Sara Galimberti, Elisa Coviello, Maria Chiara Tisi, Alessandro Morotti, Brunangelo Falini, Mauro Turrini, Agostino Tafuri, Atto Billio, Massimo Gentile, Roberto Massimo Lemoli, Adriano Venditti, Matteo Giovanni Della Porta, Francesco Lanza, Luigi Rigacci, Patrizia Tosi, Francesco Lanza, Alessandro Corso, Mario Luppi, Nicola Giuliani, Alessandro Busca, recruited participants and collected and recorded data.

All authors reviewed the manuscript and agreed with manuscript submission. All authors agreed to be accountable for all aspects of the work in ensuring that questions related to the accuracy or integrity of any part of the work are appropriately investigated and resolved.

## CONFLICTS OF INTEREST

We declare no competing financial interests.

### PEER REVIEW

The peer review history for this article is available at https://publons.com/publon/10.1002/hon.3048.

## Supporting information

Supplementary Material

Supplementary Material

## Data Availability

The data that support the findings of this study are available from the corresponding author upon reasonable request.
