# Supplementary material for: Secondary infections worsen the outcome of COVID‐19 in patients with hematological malignancies: A report from the ITA‐HEMA‐COV
Source: Hematol Oncol. 2022 Aug 12:10.1002/hon.3048. Online ahead of print. doi: 10.1002/hon.3048 (PMC9349965; doi:10.1002/hon.3048)
Supplement: Supplementary file 1 — Supplementary Material [file HON-9999-0-s002.docx]

**SUPPLEMENTAL MATERIAL**

**Supplemental Table 1** - List of isolates and respective site of isolation

|  |  | **TOTAL** |
| --- | --- | --- |
| **Urinary tract**  *Escherichia coli*  *Citrobacter braakii*  *Klebsiella pneumoniae*  *Klebsiella oxytoca*  *Klebsiella aerogenes*  *Pseudomonas aeruginosa*  *Pseudomonas fluorescens*  *Proteus mirabilis*  *Streptococcus pneumoniae*  *Enetococcus faecium*  *Enterococcus faecalis* | 8  1  6  1  1  1  1  3  3  3  3 | 32 |
| **Blood**  *Acinetobacter* spp.  *Escherichia coli*  *Klebsiella pneumoniae*  *Pseudomonas aeruginosa*  *Stenotrophomonas maltophilia*  *Corynebacterium spp*  *Cutibacterium acnes*  *Granulicatella adiacens*  *Micrococcus luteus*  *Staphylococcus aureus*  Coagulase Negative Staphylococci  *Streptococcus pneumoniae*  *Enetococcus faecium*  *Enterococcus faecalis*  Cytomegalovirus  Epstein Barr Virus  Herpes Zoster Virus  *Candida* spp. | 4  15  2  6  1  4  1  1  1  9  20  3  7  4  6  2  1  11 | 98 |
| **Bronchoalveolar lavage**  *Acinetobacter baumannii*  *Burkholderia* spp.  *Escherichia coli*  *Klebsiella oxytoca*  *Mycoplasma pneumoniae*  *Pseudomonas aeruginosa*  *Stenotrophomonas maltophilia*  *Pneumocystis jirovecii*  *Klebsiella pneumoniae*  *Staphylococcus aureus*  Cytomegalovirus  Enterovirus  Herpes Simplex 1  Rhinovirus  *Aspergillus* spp. (culture isolate and/or GM) | 1  1  3  1  1  3  1  1  1  2  6  1  1  1  5 | 29 |
| **Skin**  *Pseudomonas aeruginosa*  *Staphylococcus aureus*  Coagulase Negative Staphylococci  *Enterococcus faecalis*  Herpes Zoster Virus | 1  1  1  1  1 | 5 |
| **Feces**  *Salmonella* spp.  *Clostridium difficile* | 1  6 | 7 |
| **Other**  *Escherichia coli*  *Klebsiella pneumoniae*  *Pseudomonas aeruginosa*  *Stenotrophomonas maltophilia*  *Clostridium difficile*  *Enterococcus faecium*  *Streptococcus pneumoniae*  *Legionella* spp  *Aspergillus* spp. (serum GM) | 2  2  1  1  1  2  2  1  2 | 14 |
| **Total** |  | **185** |

**Supplemental Table 2 -** Multivariable analysis of overall survival of patients with or without secondary infection

|  | **HR** | **95%CI** | **p-value** | **Global**  **p-value** |
| --- | --- | --- | --- | --- |
| **Secondary infection** |  |  |  | <0.001 |
| No | 1 | - | - |  |
| Yes | 2.9 | 2.2-3.9 | <0.001 |  |
| **Age** | 1.0 | 1.0-1.1 | <0.001 | <0.001 |
| **COVID-19 severity** |  |  |  | <0.001 |
| Mild | 1 | - | - |  |
| Severe | 1.9 | 1.5-2.6 | <0.001 |  |
| Critical | 6.1 | 4.5-8.3 | <0.001 |  |
| **HM status** |  |  |  | 0.038 |
| CR | 1 | - | - |  |
| Not in CR | 1.4 | 1.0-1.9 | 0.038 |  |
| **HM type** |  |  |  | 0.138 |
| MPN | ref | - | - |  |
| MDS | 1.5 | 0.9-2.4 | >0.90 |  |
| AML | 1.4 | 0.9-2.2 | >0.90 |  |
| ALL-LL | 0.6 | 0.2-1.3 | >0.90 |  |
| LLC-HCL | 1.8 | 0.8-3.9 | >0.90 |  |
| LNH-LH-PCN | 0.9 | 0.6-1.3 | >0.90 |  |
