# Supplementary material for: Secondary infections worsen the outcome of COVID‐19 in patients with hematological malignancies: A report from the ITA‐HEMA‐COV
Source: Hematol Oncol. 2022 Aug 12:10.1002/hon.3048. Online ahead of print. doi: 10.1002/hon.3048 (PMC9349965; doi:10.1002/hon.3048)
Supplement: Supplementary file 2 — Supplementary Material [file HON-9999-0-s001.docx]

**APPENDIX**

**Participant Centers**

UO Ematologia, Ospedale Universitario Molinette San Giovanni Battista di Torino

UO Ematologia, IRCCS Ospedale San Raffaele, Milano

SC Ematologia, Arcispedale Santa Maria Nuova, Reggio Emilia

UO Ematologia e CTMO, ASST Spedali Civili, Brescia

UOC Ematologia, Azienda Ospedaliera Integrata di Verona

UOC Ematologia, AOU Policlinico Vittorio Emanuele, Catania

SC Ematologia, Fondazione IRCCS Policlinico San Matteo; Università degli studi di Pavia

UOC Ematologia, Azienda Ospedaliero-Universitaria Policlinico S.Orsola-Malpighi, Bologna

UOC Ematologia, ASST Sette Laghi, Osp. Di Circolo e Fon. Macchi Varese

UOC Ematologia, Fondazione IRCCS Ca' Granda Ospedale Maggiore Policlinico, Milano

IFO - Ematologia e Trapianto Cellule -Istituto Nazionale del Cancro di Regina Elena, Roma

SC Ematologia Ospedale S. Croce di Cuneo

SC Ematologia, Istituto Nazionale dei Tumori, Milano

SC Ematologia, Ospedale Niguarda Milano

UO Ematologia, Ospedale dell'Angelo di Mestre, Mestre-Venezia

S.O.D. Ematologia, Azienda Ospedaliero Universitaria Careggi, Firenze

UOC Ematologia, Ospedale S. Gerardo di Monza

UOC Ematologia, Policlinico Bari

UOC Ematologia, Ospedale Civili Ca’ Foncello, Treviso

UOC, Ematologia Azienda Ospedaliero Universitaria Arcispedale S. Anna, Ferrara

UOC Ematologia Istituto Nazionale Tumori - IRCCS "Fondazione G. Pascale", Napoli

UOC Ematologia Azienda Ospedaliera di Padova

UOC Ematologia, Policlinico Santa Maria alle Scotte, Siena

UOC Ematologia, AOU Pisana- Santa Chiara, Pisa

UOC Ematologia, Ospedale Policlinico San Martino, Genova

UOC Ematologia, Ospedale San Bortolo, Vicenza

UOC Ematologia, Ospedale san Luigi Gonzaga Orbassano

UOC Ematologia e Trapianto di Midollo Osseo, Azienda Ospedaliera di Perugia

UOC Ematologia, Ospedale Valduce, Como

UOC Ematologia, AOU Azienda Ospedaliera Universitaria Sant'Andrea, Roma

UOC Ematologia e Centro Trapianto Midollo Osseo, Ospedale di Bolzano

UOC Ematologia, Azienda Ospedaliera di Cosenza

UOC Ematologia, Ospedale Policlinico S.Martino IRCCS – Genova

UOC Ematologia, Policlinico Tor Vergata, Roma

UO Ematologia, Istituto Clinico Humanitas, Rozzano

UO Ematologia dell'Ospedale Santa Maria delle Croci, Ravenna

UO Ematologia e Trapianti di Cellule Staminali, A.O. S. Camillo-Forlanini, Roma

UO Ematologia, Ospedale Infermi di Rimini

UOC Ematologia, Azienda Sanitaria Universitaria Giuliano Isontina, Trieste

UOC di Ematologia, Ospedale di Legnano, Legnano

UOC di Ematologia, Policlinico di Modena

UOC Ematologia e CTMO, Azienda Ospedaliero-Universitaria di Parma

Clinica Ematologia, Azienda Ospedaliera-Universitaria Santa Maria della Misericordia, Udine

SSD Ematologia, Ospedale degli Infermi, Biella

UOC Ematologia, IEO, Milano

UOC Servizio di Ematologia e Medicina Trasfusionale, Ospedale Luigi Sacco, Milano

IRCCS Opera di San Pio da Pietralcina - San Giovanni Rotondo

UOC di Ematologi, Ospedale C. Massaia Asti

UOC Ematologia, I.R.C.C.S Istituto Tumori Giovanni Paolo II, Bari

UOC Ematologia Ospedale SS Antonio e Biagio e Cesare Arrigo di Alessandria

UOC Ematologia- Azienda Ospedaliera San Giovanni Addolorata, Roma

UO Ematologia e CTMO, Azienda Ospedaliera Ospedali Riuniti Pesaro

UOC Ematologia Policlinico Umberto I, Roma

[UOC Ematologia, Azienda Ospedaliero-Universitaria Maggiore della Carità di Novara](https://trasparenza.maggioreosp.novara.it/)

UOC Ematologia e trapianto di cellule staminali, Policlinico Universitario Campus Bio-Medico, Roma

SCDU Ematologia e terapie cellulari, Ospedale Mauriziano Umberto I, Torino

UOC Ematologia e Terapia Cellulare, Ospedale Mazzoni, Ascoli Piceno

UOC Onco-Ematologia ASST Valle Olona, Busto Arsizio

UO Ematologia e CTMO, ASST Cremona

UOC Ematologia, Ospedale Santa Maria Goretti, Latina

UOC Ematologia e Trapianti di Midollo, AOU Federico II, Napoli

SC Ematologia, Fondazione Policlinico Universitario Agostino Gemelli IRCCS, Università Cattolica Roma
